# Interactions with M Cells and Macrophages as Key Steps in the Pathogenesis of Enterohemorragic *Escherichia coli* Infections

**DOI:** 10.1371/journal.pone.0023594

**Published:** 2011-08-17

**Authors:** Lucie Etienne-Mesmin, Benoit Chassaing, Pierre Sauvanet, Jérémy Denizot, Stéphanie Blanquet-Diot, Arlette Darfeuille-Michaud, Nathalie Pradel, Valérie Livrelli

**Affiliations:** 1 Clermont Université, Université d'Auvergne, Centre de Recherche en Nutrition Humaine Auvergne, JE 2526 Evolution des bactéries pathogènes et susceptibilité génétique de l'hôte, Clermont-Ferrand, France; 2 INRA, Institut National Recherche Agronomique, Unité Sous Contrat USC-2018, Clermont-Ferrand, France; 3 Clermont Université, Université d'Auvergne, Centre de Recherche en Nutrition Humaine Auvergne, ERT 18, Conception, Ingénierie et Développement de l'Aliment et du Médicament, Clermont-Ferrand, France; 4 Clermont Université, Université d'Auvergne, UFR Pharmacie, Clermont-Ferrand, France; 5 CHU Clermont Ferrand, Pôle des Pathologies Digestives, Clermont-Ferrand, France; 6 CHU Clermont-Ferrand, Service Bactériologie Mycologie Parasitologie, Clermont-Ferrand, France; Indian Institute of Science, India

## Abstract

Enterohemorrhagic *Escherichia coli* (EHEC) are food-borne pathogens that can cause serious infections ranging from diarrhea to hemorrhagic colitis (HC) and hemolytic-uremic syndrome (HUS). Translocation of Shiga-toxins (Stx) from the gut lumen to underlying tissues is a decisive step in the development of the infection, but the mechanisms involved remain unclear. Many bacterial pathogens target the follicle-associated epithelium, which overlies Peyer's patches (PPs), cross the intestinal barrier through M cells and are captured by mucosal macrophages. Here, translocation across M cells, as well as survival and proliferation of EHEC strains within THP-1 macrophages were investigated using EHEC O157:H7 reference strains, isogenic mutants, and 15 EHEC strains isolated from HC/HUS patients. We showed for the first time that *E. coli* O157:H7 strains are able to interact *in vivo* with murine PPs, to translocate *ex vivo* through murine ileal mucosa with PPs and across an *in vitro* human M cell model. EHEC strains are also able to survive and to produce Stx in macrophages, which induce cell apoptosis and Stx release. In conclusion, our results suggest that the uptake of EHEC by M cells and underlying macrophages in the PP may be a critical step in Stx translocation and release *in vivo*. A new model for EHEC infection in humans is proposed that could help in a fuller understanding of EHEC-associated diseases.

## Introduction

Enterohemorrhagic *Escherichia coli* (EHEC), a subset of Shiga toxin-producing *E. coli* (STEC), have been associated with human diseases, ranging from uncomplicated diarrhea to hemorrhagic colitis (HC) and hemolytic-uremic syndrome (HUS). A recent major outbreak in Germany, with thousands cases of foodborne illness (and approximately 25% of them progressing to HUS) has shed light on EHEC [1], [2]. The gastrointestinal tract of cattle and other ruminants appears to be the main reservoir of STEC strains [3], [4]. Several studies have reported a high prevalence of STEC belonging to a wide range of serotypes in animals and food products [3], [4], [5]. However, only a limited number of serotypes have been associated with human disease, among which O157:H7 is predominant [3], [6]. The association of serotypes with disease of varying severity in humans and with outbreaks or sporadic disease has led to the proposal that STEC be classified into 5 seropathotypes, from A (most virulent) to E (serotypes that have not been involved in disease in humans) [7].

EHEC colonize the digestive tract of humans and produce Shiga toxins (Stx1 and/or Stx2), also known as Verotoxins, which are essential for virulence. Epidemiological studies, together with *in vitro* and *in vivo* experiments, have shown that Stx2 is the most common virulence factor associated with severe human disease [3]. Stx are composed of an enzymatically active A subunit and a pentameric B subunit. The B subunits form a doughnut-shaped structure with a central pore and bind to the glycosphingolipid globotriaosylceramide (Gb3, also known as CD77), which is expressed at the surface of endothelial cells, leading to subsequent internalization of the toxin [8]. The A subunit is able to inhibit elongation of the peptide chain during protein synthesis, resulting in eukaryotic cell death, tissue damage and organ failure [3], [8]. EHEC, together with enteropathogenic *E. coli* (EPEC), belong to the attaching and effacing (A/E) bacterial pathogens. They induce histopathological lesions characterized by localized effacement of the brush border microvilli and intimate attachment of the bacteria to the apical membrane of epithelial cells, through the formation of cytoskeletal actin pedestals [9], [10]. All the genes involved in A/E lesions map to a pathogenicity island, the Locus for Enterocyte Effacement (LEE) that encodes a type III secretion system (TTSS) involved in the injection of several proteins and effectors [11]. The first gene associated with A/E lesions is the *eae* gene encoding intimin, an outer membrane protein. Intimin binding to Tir, a receptor translocated from the bacteria to the host cell, mediates intimate adherence of the bacterium to epithelial cells. Several intimin types have been identified that may determine the host tropism [12]. Many other factors have been suggested to be associated with EHEC virulence. These include enterohemolysin, a pore-forming cytolysin, an extracellular serine-protease, and a catalase-peroxidase [3], [13]. Flagella are also thought to play a role in adherence to epithelial cells. H7 flagella have been shown to act as an adhesin to bovine intestinal epithelium [14] and H7 flagellin (encoded by the *fli*C gene) induced proinflammatory signals in human colon epithelial cells through activation of the MAP kinase and NF-κB pathways [15].

Experiments using *in vitro* organ culture (IVOC) of human intestinal mucosa revealed a preferential tropism of EHEC O157:H7 for the follicle-associated epithelium (FAE) overlying the distal ileal Peyer's patches (PPs), where it causes A/E lesions [12], [16], [17]. The FAE is characterized by the presence of specialized “membranous” or “microfold” cells (M cells), which are specialized in the translocation of microorganisms and antigens from the intestinal lumen to the basolateral side of the epithelium, where they are delivered to the underlying macrophages [18], [19]. While M cells are primarly involved in sampling intestinal antigens, many invasive *Enterobacteriaceae*, such as *Salmonella enterica* serovar Typhimurium [20], rabbit enteroadherent *E. coli* [21] or Adherent-Invasive *E. coli* (AIEC) isolated from patients with Crohn's disease [22], [23], take advantage of the transcytotic characteristics of M cells to use them as an entry site to translocate across the intestinal barrier.

Systemic complications associated with EHEC diseases such as HUS require the expression and translocation of Stx, which are produced by colonizing bacteria, from the gut lumen to underlying tissues and the bloodstream. However, the mechanism of Stx translocation across the epithelial barrier remains unclear as human intestinal cells lack Gb3, the Stx receptor. Since EHEC O157:H7 was found to interact initially with FAE in both humans and cattle [12], [16], [24], we hypothesized that the uptake of bacteria by M cells and underlying macrophages may be the first stage in Stx translocation, and may represent an important step in the pathogenesis of EHEC infections. We report here the interactions of EHEC strains *in vivo* and *ex vivo* with murine PPs, and *in vitro* using a human M cell model. Binding to the FAE and translocation through M cells may result in the rapid contact of bacteria with underlying human macrophages. However, little information is known about the interactions between EHEC strains and these cells. We decided, therefore, to investigate entry, survival and proliferation of EHEC strains belonging to seropathotypes A to C in human THP-1 macrophages. EHEC were found to survive and to produce Stx within macrophages, leading to host cell apoptosis and Stx release.

## Materials and Methods

### Bacterial strains

Bacterial strains and plasmids used in this study are given in **Table 1**. The green fluorescent protein (GFP)-expressing strains were obtained by electroporation of a high-copy plasmid pFPV25.1. Before use, each strain was streaked onto LB agar plates (BD Bioscience, USA) and grown overnight at 37°C in LB broth, unless otherwise stated. When necessary, kanamycin or ampicillin was added to the medium at 50 µg/ml. For *ex vivo* experiments, a non pathogenic *E. coli* strain MG1655 (Rif^R^) was used and plated on LB medium containing rifampicin (300 µg/ml).

**Table 1:** Bacterial strains and plasmid used in this study.

Strains or plasmids	Serotype	Seropathotype	Characteristics	*stx* genotype	Source or reference
**Strains**					
86-24 WT	O157:H7	A	Enterohemorragic *E. coli* O157:H7 reference strain	*stx1− stx2+*	[44]
86-24 Δ*stx2*	O157:H7	NA	86-24 isogenic mutant with *stx2* gene deleted	*stx1− stx2−*	[15]
86-24 Δ*eae*	O157:H7	NA	86-24 isogenic mutant with *eae* gene deleted	*stx1− stx2+*	[15]
86-24 Δ*fliC*	O157:H7	NA	86-24 isogenic mutant with *fliC* gene deleted	*stx1− stx2+*	[15]
EDL933	O157:H7	A	Enterohemorragic *E. coli* O157:H7 reference strain	*stx1+ stx2+*	ATCC 43895
CHVi-1	O157:H7	A	*E. coli* isolated from a clinical case with HUS	*stx1− stx2+*	[13]
CH1898	O157:H7	A	*E. coli* isolated from a clinical case with HUS	*stx1− stx2+*	[13]
CH075	O157:H7	A	*E. coli* isolated from a clinical case with HUS	*stx1− stx2+*	[13]
CH087	O103:H2	B	*E. coli* isolated from a clinical case with HUS	*stx1+ stx2−*	[13]
CH089	O103:H2	B	*E. coli* isolated from a clinical case with HC	*stx1+ stx2−*	[13]
NV-10	O26:H11	B	*E. coli* isolated from a clinical case with diarrhea	*stx1+ stx2−*	[4]
CH071	O157:H26	C	*E. coli* isolated from a clinical case with HUS	*stx1− stx2+*	[13]
CH013	O91:H10	C	*E. coli* isolated from a clinical case with HUS	*stx1− stx2+*	[13]
CH085	O91:H10	C	*E. coli* isolated from a clinical case with HUS	*stx1− stx2+*	[13]
CH014	O91:H21	C	*E. coli* isolated from a clinical case with HUS	*stx1− stx2+*	[13]
VTH13	O91:H21	C	*E. coli* isolated from a clinical case with HC	*stx1+ stx2+*	Blanco J^a^
CH016	O174:H−	C	*E. coli* isolated from a clinical case with HUS	*stx1− stx2+*	[13]
CH123	O5:H−	ND	*E. coli* isolated from a clinical case with HC	*stx1+ stx2−*	[13]
CH017	O+:H−	ND	*E. coli* isolated from a clinical case with HUS	*stx1− stx2+*	[13]
CH023	O+:H−	ND	*E. coli* isolated from a clinical case with HUS	*stx1− stx2+*	[13]
LF82	O83:H1	NA	Adherent-Invasive *E. coli* isolated from a patient with Crohn's disease	NA	[45]
MG1655	OR:H48/K−	NA	Non pathogenic *E. coli*	NA	Laboratory stock
C600	K-12	NA	Non pathogenic *E. coli*	NA	Laboratory stock
**Plasmid**					
pFPV25.1			Plasmid constitutively expressing GFP		[46]

NA, not applicable; ND, not determined; HUS, Hemolytic-uremic syndrome; HC, Hemorrhagic colitis; WT, wild-type.

a Laboratorio de Referencia de *E. coli*, Universidade de Santiago de Compostela, Lugo, Spain.

### Ethics statement

Animal protocols were approved by the committee for ethical issues, CEMEA Auvergne (Agreement to Nicolas Barnich, CEMEA CE16-09, Clermont-Ferrand, France). Mice were killed by cervical dislocation according to animal care procedure.

### *Ex vivo* interactions with murine Peyer's patches

Eight- to ten-week old FVB/N wild-type male mice were bread and reared in the animal care facility at the Université d'Auvergne (Clermont-Ferrand, France) under specific pathogen free (SPF) conditions.

Biopsies from ileum with or without PP were immediately removed from mouse intestine and used for Ussing chamber experiments. Briefly, 1 cm of ileal samples was opened along the mesenteric border, splatted and mounted in an Ussing chamber with an opening area of 0.96 mm^2^. Tissues were bathed in 37°C-oxygenated Ringer solution for 5 h. A total of 1.6 ml of 1×10^7^ CFU/ml (colony forming units) of EHEC or non pathogenic *E. coli* was added to the mucosal compartment and 1.6 ml of Ringer buffer was placed into the serosal side. To quantify the bacteria translocated across mucosa, medium from the serosal compartment was collected every hour, diluted, and plated onto LB agar plates. The integrity of ileal mucosa was monitored throughout Ussing chamber experiments using Fluorescein isothiocyanate (FITC) (Sigma, St. Louis, MO). FITC diffusion was assessed by fluorescence measurement in a microplate fluorescent reader (Fluoroskan Ascent FL, Thermo) at an excitation wavelength of 485 nm and an emission wavelength of 522 nm.

### Mice ileal loop assay

Interactions of EHEC bacteria with PPs were studied using mouse ileal loops, as previously described by Hitotsubashi *et al.* [25]. FVB/N wild-type male mice were starved for 24 h before operation but provided with water *ad libitum*. The animals were anesthetized and their intestine exteriorized through a midline incision. Two or three intestinal segments (about 1 cm), each containing one PP, were ligated, and 5×10^8^ CFU of bacteria (EHEC 86-24 WT and 86-24 Δ*stx_2_*, a non-pathogenic *E. coli* strain K-12 C600, and a control AIEC strain LF82) were injected into the ileal loop. Two hours after injection, the animals were killed and the loops were excised. To find a colocalization of 86-24 WT bacteria with M cells, 4 µm sections of paraffin-embedded PPs were stained with Tetramethyl Rhodamine Isocyanate (TRITC)-labeled *Ulex europaeus* agglutinin 1 (UEA 1, Sigma-Aldrich, France) to label M cells. Hoechst was used for nuclear staining and a monoclonal mouse antibody raised against *E. coli* LPS O157 (Abcam, France) was used to label EHEC bacteria. The slides were then analyzed with a Zeiss LSM 510 Meta confocal microscope.

### Cell lines and cell culture

The *in vitro* M cell co-culture model was first developped by Kerneis *et al.* and later adapted by Gullberg *et al.* [26], [27]. The human colorectal adenocarcinoma cell-line Caco-2 clone 1 [27] was grown in complete DMEM (PAA, Austria), supplemented with 10% heat-inactivated fetal bovine serum (FBS) (Lonza, Switzerland), 4 mM L-glutamine (PAA), 100 U/ml penicillin (PAA) and 100 µg/ml streptomycin (PAA). The human Burkitt's lymphoma cell-line Raji B (ECACC 85011429) was grown in complete RPMI-1640 medium (PAA), supplemented with 10% heat-inactivated FBS, 8 mM L-glutamine, 100 U/ml penicillin and 100 µg/ml streptomycin.

The human (macrophage-like) monocyte cell line THP-1 (ATCC TIB202) was maintained in RPMI-1640 medium supplemented with 10% heat-inactivated FBS and 4 mM L-glutamine. THP-1 cells were activated using 20 ng/ml phorbol 12-myristate 13-acetate (PMA) (Sigma-Aldrich), seeded in 24-well tissue culture plates (BD Falcon, USA) at a density of 5×10^5^ cells per cm^2^, and grown for 18 h.

Vero cells (African green monkey kidney cells, ATCC CRL 1587) were grown at 37°C in Eagle basal medium (Seromed, Germany) supplemented with 10% FBS, 8 mM l-glutamine, 100 U/ml penicillin,100 µg/ml streptomycin and 1% vitamin solution, and seeded in 96-well tissue culture plates at a density of 10^6^ cells per ml.

All the cell lines were grown at 37°C under 5% CO_2_ in a humidified atmosphere.

### Vero toxin assay

To quantify Stx production, bacterial culture supernatants and infected THP-1 supernatants were tested for cytotoxicity in the Vero cell assay, as previously described [4]. THP-1 cells were infected for 2 h, extra-cellular bacteria were removed and fresh medium was added for 3 h, before testing Stx in supernatants. The Stx titre was expressed as the reciprocal of the highest filtrate dilution that caused 50% cell detachment after 24 h of incubation, as judged by the dye intensity and by microscopic observation (the breakpoint for a positive result was a titer of 4). Known concentrations of purified Stx2 (Toxin Technology, USA) were used to estimate the range of Stx2 release within macrophages. Each experiment was performed at least 3 times.

### Bacterial translocation across M cells

A total of 1×10^6^ Caco-2-cl1 cells per ml were seeded onto the apical aspect of Transwell™ filters (Millipore Ltd, UK) previously coated with BD Matrigel™ (USA). Cells were carefully cultured for 17 days until they reached a fully differentiated phenotype. 5×10^5^ Raji-B cells were added to the basolateral compartment of Caco-2 monolayers and co-culture was maintained for 4–6 days. Monocultures of Caco-2 cells on matched filter supports were used as control.

For translocation assay, apical surface of M-cells were infected with 1×10^7^ bacteria per transwell. Samples from basolateral media were collected every hour for 5 h, and 10-fold dilutions were plated onto LB agar. The integrity of cell monolayers was tested by monitoring trans-epithelial electrical resistance (TEER) with a Millicell®-ERS (Millipore).

### Bacterial uptake, survival and replication in human macrophages

Bacterial uptake, survival and replication were measured by the gentamicin protection assay. Bacterial strains were grown for 2 h at 37°C in LB. Before infection, cell monolayers were washed twice with PBS and the medium was replaced with 1 ml of RPMI-1640 supplemented with 10% heat-inactivated FBS for 2 h. THP-1 cells were infected with a multiplicity of infection (MOI) of 10 bacteria per macrophage. After a 2-h incubation, infected macrophages were washed twice with PBS, and fresh cell culture medium containing 20 µg/ml of gentamicin was added to kill extracellular bacteria. After incubation for 1, 4, 24 or 48 h, the medium was removed, cells were washed once with PBS, and a 5 min treatment with 500 µl 1X-triton was used to lyse the eukaryotic cells. This concentration of Triton X-100 had no effect on bacterial viability for at least 30 min. Samples were collected, diluted, and plated onto Mueller-Hinton agar plates to determine the number of bacteria surviving gentamicin killing assay. Survival was expressed either as CFU/well or as the mean percentage of the number of bacteria recovered at 4, 24 and 48 h post-infection, compared to that at 1 h post-infection, defined as 100%. Each experiment was performed at least five times.

### Lactate dehydrogenase activity (LDH)

Supernatants of infected macrophages were sampled at 1, 4, 8 and 24 h of gentamicin treatment, and assayed for LDH activity using reduced nicotinamide adenine dinucleotide (NAD) as a substrate (LDH kit, Boehringer Mannheim, France).

### Hoechst Staining of THP-1 macrophages

Coverslips with adherent infected THP-1 cells were collected at specified time points, fixed with 4% paraformaldehyde, washed twice with PBS, and stained with Hoechst (5 mg/ml) for 30 min at room temperature. Coverslips were washed three times with saponin, twice with PBS, and mounted on glass slides. Apoptotic nuclei were quantified by fluorescence microscopy. Each experiment was performed four times. Five random fields were counted on each slide.

### Transmission electron microscopy (TEM)

Cross sections of THP-1 cells were prepared as follows. After infection, cells were fixed with 3% glutaraldehyde in 0.2 M cacodylate buffer at 4°C for 2 h and post fixed in 1% OsO_4_ in cacodylate buffer at 4°C for 1 h. After dehydratation in ethanol, cultures were embedded in a 2-mm-thick Epon coating and polymerized for 3 days at 60°C. Suitable areas were oriented parallel to the cell layer surface on Epon blocks with an Epon mixture. Ultrathin sections were contrasted with uranyl acetate and lead citrate. Grids were examined with Hitachi H7650 TEM.

### Confocal microscopy of infected THP-1 macrophages

After infection with GFP-expressing bacteria, as described above, THP-1 cells were washed with PBS to eliminate extracellular bacteria and fixed with 3% paraformaldehyde for 10 min. Fixed-cells were washed with PBS, incubated for 5 min with 0.1 M glycine, washed with PBS, and permeabilized with 0.1% Triton X-100 for 20 min. After PBS-washing, slides were incubated twice for 10 min, each time with PBS–0.2% gelatin. Actin cytoskeleton was stained for 15 min using TRITC-phalloidin (Sigma-Aldrich, France). Monolayers were then washed with PBS and distilled water, stained with 49,6-diamidino-2-phenylindole (DAPI) and mounted on glass slides with a Mowiol solution (Calbiochem, Darmstadt, Germany). The slides were examined with a Zeiss LSM 510 Meta confocal microscope.

### Statistical analysis

The Student *t*-test was used for unpaired data with a 5% level of significance for the comparison of values. For Ussing chamber experiments, comparisons were made with the unpaired Mann Whitney test.

## Results

### EHEC strains interact *ex vivo* with murine Peyer's patches and *in vivo* with murine M cells

**Figure 1:**
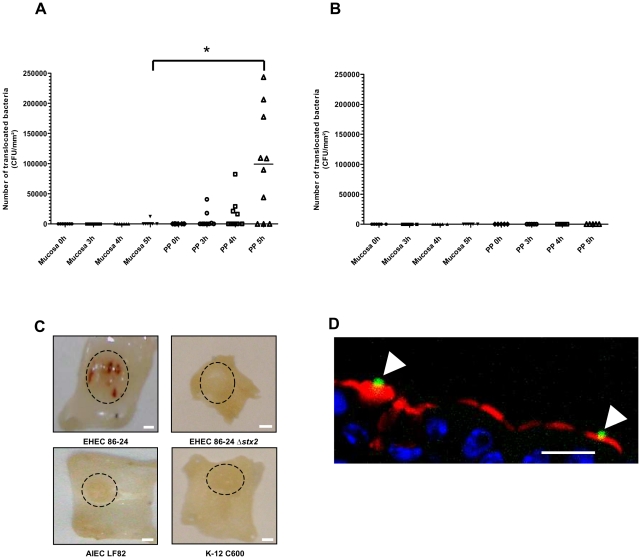
*Ex vivo* transmucosal uptake of EHEC bacteria by murine Peyer's patches, and *in vivo* interactions with murine M cells in ileal loop assays. (**A**) *Ex vivo* interaction of *E. coli* O157:H7 (strain 86-24 WT) with murine mucosa isolated from the ileum with (*n = 10*) or without (*n = 7*) Peyer's patches (PPs) in Ussing chambers. Each point represents the number of translocated bacteria (CFU/mm^2^) for one experiment at the time of infection (0 h) and after 3 h, 4 h and 5 h of contact. Lines represent the median values of the group. Comparisons were made with the Mann-Whitney test. *, Ileum with PP significantly different from ileal mucosa (*P*<0.05). (**B**) *Ex vivo* interaction of non pathogenic *E. coli* MG1655 with murine ileal mucosa isolated from the ileum with (*n = 5*) or without (*n = 6*) PPs in Ussing chambers. Each point represents the number of translocated bacteria (CFU/mm^2^) for one experiment at the time of infection (0 h) and after 3 h, 4 h and 5 h of contact. (**C**) Macroscopic analysis of murine PP sections after a two-hour contact with EHEC 86-24 WT, 86-24 Δ*stx2*, AIEC strain LF82 and non pathogenic *E. coli* K-12 strain C600 in ileal loop assays. Dashed circles indicate PP. Scale bar = 1 mm. (**D**) Confocal analysis of murine PP sections after labeling of EHEC 86-24 WT with anti LPS O157 antibody (green), of M cell with UEA-1 TRITC (red) and DNA with Hoechst (blue). Scale bar = 20 µm. Arrowheads, bacteria associated with UEA-1-positive cells.

EHEC mucosal translocation across murine ileal mucosa was studied in Ussing chambers. Transmucosal uptake of EHEC 86-24 WT bacteria through murine ileal mucosa without PP was very low, even after 5 h of infection (Figure 1A). In contrast, bacterial translocation was observed for ileal biopsies containing PP, reaching a median number of translocating bacteria of 1.1×10^5^ CFU/mm^2^ at 5 h, versus no translocating bacteria for 6 out of 7 ileal mucosa without PP (*P*<0.05) (Figure 1A). As a control, a non pathogenic *E. coli* strain was not able to translocate across ileal mucosa either with or without PP (Figure 1B). The integrity of ileal mucosa was monitored throughout Ussing chamber experiments: FITC diffusion was limited and there was no difference between Stx-producing bacteria and non pathogenic *E. coli* (Figure S1). To confirm *ex vivo* data, *in vivo* experiments using mouse ileal loop containing one PP were performed. Interestingly, bloodshot PPs were observed macroscopically in ileal mucosa after a two-hour contact with wild type EHEC. This was not the case with a Stx-negative *E. coli* strain 86-24 Δ*stx_2_*, nor with the AIEC strain LF82, an invasive *E. coli* pathovar known to target PPs, nor with a non pathogenic *E. coli* strain K-12 C600 (Figure 1C). Confocal analysis revealed that 86-24 WT EHEC bacteria interact with murine M cells (Figure 1D).

### EHEC strains are able to translocate across M cells

**Figure 2:**
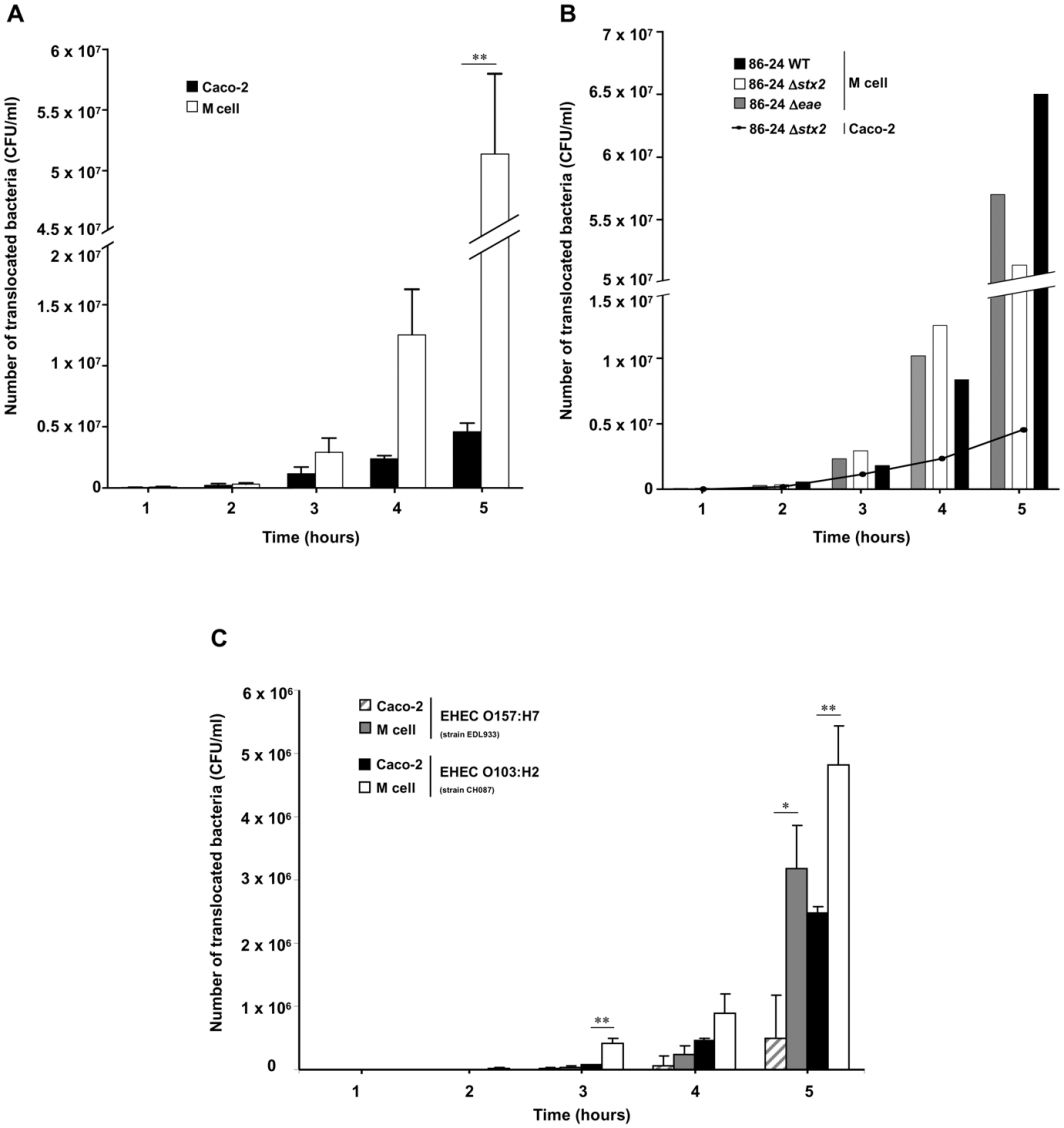
*In vitro* translocation of EHEC strains across human M cell monolayers. The number of translocated bacteria (CFU/ml) at 1 to 5 h post infection was determined in M cell monolayers *versus* control Caco-2 monolayers, for EHEC 86-24 Δ*stx2* (O157:H7) (**A**) and compared with 86-24 WT and 86-24 Δ*eae* (**B**) and with EDL 933 (O157:H7) and CH087 (O103:H2) (**C**). Results are means ± standard error of mean (SEM) for replicate experiments. Translocation through M cell monolayers significantly different from that of Caco-2 monolayers (* *P*<0.05 or ** *P*<0.01).

Since 86-24 WT EHEC interact *in vivo* with murine M cells, we used an *in vitro* model in which human intestinal epithelial cells acquire M cell-like characteristics after being co-cultured with Raji B cells to study bacterial translocation. Experiments were first performed using an *E. coli* 86-24 Δ*stx_2_* mutant to avoid possible cell death induced by Stx. Translocation of *E. coli* 86-24 Δ*stx_2_* remained at low levels with the control Caco-2 monolayer [27] (4.6×10^6^±1×10^6^ CFU/ml), but increased with time in the *in vitro* M cell model, reaching 5.1×10^7^±1.7×10^7^ CFU/ml at 5 h post infection (*P*<0.01) (Figure 2A). The behavior of 86-24 WT and 86-24 Δ*eae* mutant was then analyzed and was similar to that of 86-24 Δ*stx_2_* (Figure 2B), indicating that (i) Stx does not interfere with the co-culture model and (ii) intimin (product of the *eae* gene) is not directly involved in translocation in this model. An additional O157:H7 reference strain (EDL933) and a non-O157 EHEC of serotype O103:H2 (CH087) were also able to translocate across M cells (Figure 2C), supporting the hypothesis that this mechanism could be extended to all EHEC strains. In contrast, the non pathogenic *E. coli* strain K-12 C600 was not able to translocate at levels similar to that of EHEC strains, since only 7.4×10^4^ CFU/ml were recovered after a 5 h contact with M cells. The translocation of EHEC bacteria was not the result of a loss of the monolayer integrity, since TEER stayed constant during the 5 h of infection (Figure S2).

### EHEC strains can replicate within THP-1 macrophages

The study was performed with 15 EHEC strains isolated from HC/HUS patients and two O157:H7 reference strains (86-24 WT and EDL933). Bacterial uptake, survival and replication in THP-1 cells were measured by the gentamicin protection assay. The non pathogenic *E. coli* strain K-12 C600 was used as a negative control and AIEC strain LF82 (isolated from a patient with Crohn's disease and able to highly replicate within macrophages) as a positive control [28], [29]. The number of intracellular bacteria was determined 1 h, 24 h and 48 h post-infection in the presence of gentamicin. The ability of EHEC strains to enter macrophages varied widely from one strain to another. The amount of intracellular bacteria at 1 h post-infection ranged from 1.7×10^4^ CFU/well for CH016, to 5.8×10^5^ CFU/well for CH015 (data not shown). The ability to enter macrophages was not related to serotype or belonging to a seropathotype.

**Figure 3:**
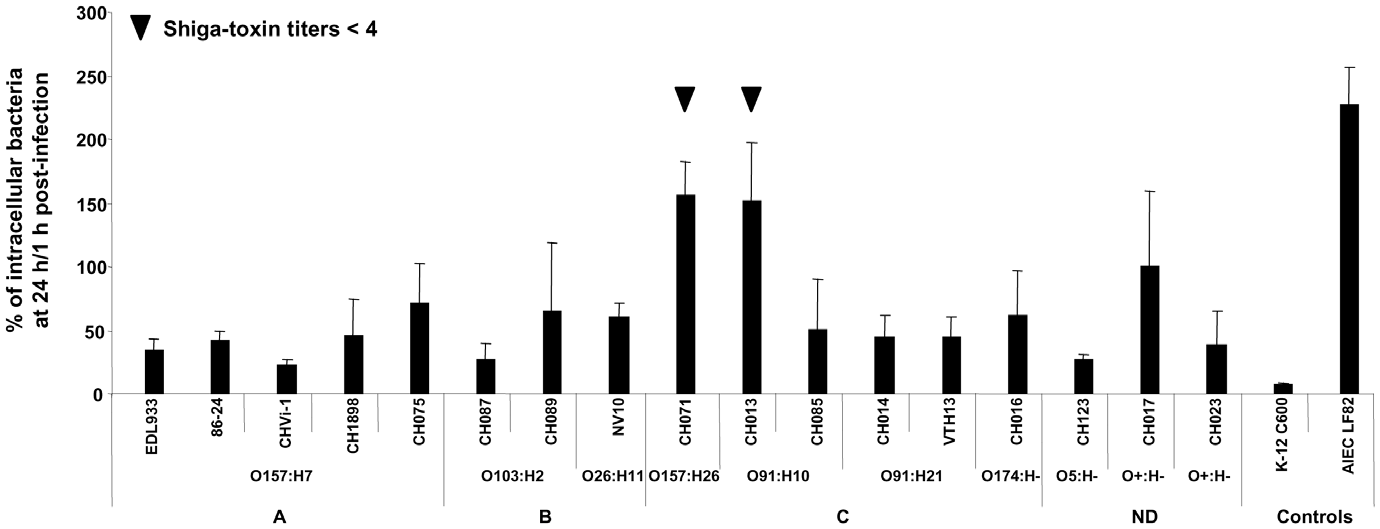
Survival and replication of EHEC strains within human THP-1 macrophages at 24 h post-infection. Results are expressed as the number of intracellular bacteria at 24 h relative to that obtained at 1 h after gentamicin exposure, taken as 100%. A, seropathotype A; B, seropathotype B; C, seropathotype C; ND, seropathotype not determined. All assays were performed independently at least five times. Results are means ± Standard Deviation (SD) for the replicate experiments.

Survival or replication of EHEC strains at 24 h post-infection was expressed as a percentage compared to the number of bacteria at 1 h, defined as 100%. The nonpathogenic *E. coli* K-12 C600 control strain was slowly but efficiently killed following phagocytosis by THP-1 cells: only 7% of the bacteria initially internalized were recovered at 24 h post-infection, evidence of the bactericidal activity of the THP-1 macrophages (Figure 3). All EHEC strains were able to survive within macrophages after 24 h. Compared to those of the negative control (*E. coli* K-12 C600), the percentages of survival were higher, ranging from 23% for CHVi-1 to 155% for CH071. However, in contrast with what was observed for the positive control (AIEC strain LF82), the number of intracellular bacteria at 24 h post-infection was smaller than at 1 h post-infection (less than 100%) in all but two strains (CH013 and CH071). No link was found between the ability of strains to survive/replicate and their serotype. After investigating the ability of strains to express Stx, we observed that the strains able to replicate within macrophages no longer expressed Stx (Stx titers<4, see Figure 3).

**Figure 4:**
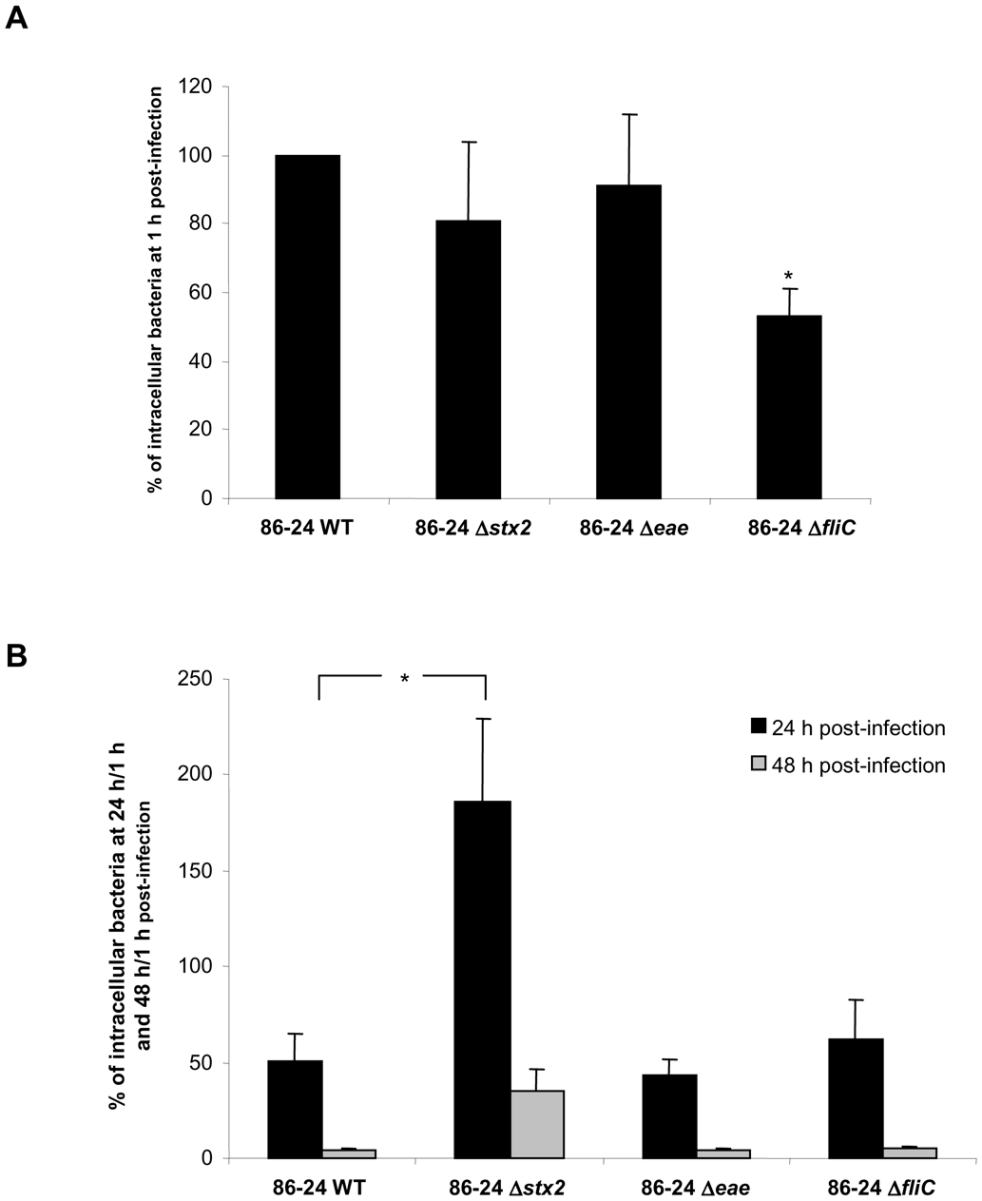
Entry and survival of EHEC strains within THP-1 macrophages. Percentage of intracellular bacteria at 1 h relative to 86-24 WT taken as 100% (**A**), and percentage of intracellular bacteria at 24 h/1 h and 48 h/1 h post-infection (**B**) for EHEC 86-24 WT, and 86-24 Δ*stx*2, 86-24 Δ*eae*, and 86-24 Δ*fli*C isogenic mutants. All assays were performed independently at least four times. Results are means ± SD for the replicate experiments. Panel A) * 86-24 Δ*fliC* significantly different from 86-24 WT (*P*<0.05). Panel B) * 86-24 Δ*stx*2 significantly different from 86-24 WT at 24 h post-infection (*P*<0.05).

To confirm that Stx play a role in the ability of EHEC strains to survive and/or replicate within macrophages, *stx* isogenic mutants of the O157:H7 reference strains 86-24 WT and EDL933 were used. For 86-24 WT, the number of bacteria internalized after 1 h, defined as 100%, was 2.1×10^5^ CFU/well (Figure 4A), and the survival rate was 51%, indicating that the level of intracellular bacteria was two-fold lower after 24 h than after 1 h (Figure 4B). The entry of the 86-24 Δ*stx*_2_ mutant was slightly less than that of the wild type (81%, Figure 4A), but the percentage of survival was 186% (Figure 4B), indicating that the number of intracellular bacteria had almost doubled compared to time 1 h. The behavior of the isogenic Δ*eae* mutant did not differ from that of the 86-24 WT strain. Interestingly, the percentage of survival at 48 h post-infection was low for all the strains tested, around 5% for Stx-producing strains and 35% for Δ*stx*_2_ mutants and non-Stx-producing bacteria. Identical results were obtained with EDL933 and its isogenic Δ*stx* and Δ*eae* mutants (data not shown).

### Cytotoxic effects of EHEC infection on THP-1 macrophages

To check if EHEC were able to produce Stx within macrophages, supernatants of infected THP-1 cells were tested using a Vero toxin assay. High amounts of Stx were recovered from THP-1 macrophages infected with EHEC 86-24 WT and EDL933: the cytotoxic titer was 1/256 (1/2^8^) and the average amount of toxin was estimated to be 8.2±1.8 ng/ml. As expected, no cytotoxic effect was observed in the supernatants of THP-1 infected with a non pathogenic *E. coli* strain or the 86-24 Δ*stx*2 mutant (Table 2).

**Table 2:** Quantification of Stx released after THP-1 infections.

Strains	Cytotoxic titer^a^	Stx concentration^b^ (ng/ml)
86-24 WT	1/256	8.2±1.8
86-24 Δ*stx2*	^-^	0
86-24 Δ*eae*	^-^	0
EDL933	1/256	8.2±1.8
K-12 C600	^-^	0

a The verotoxin titer was expressed as the reciprocal of the highest sample dilution which caused 50% cell detachment after 24 h of incubation.

b Stx concentration in the supernatant of infected THP-1 macrophages.

The results of 3 independent sets of data are presented.

-, no cytotoxic effect.

**Figure 5:**
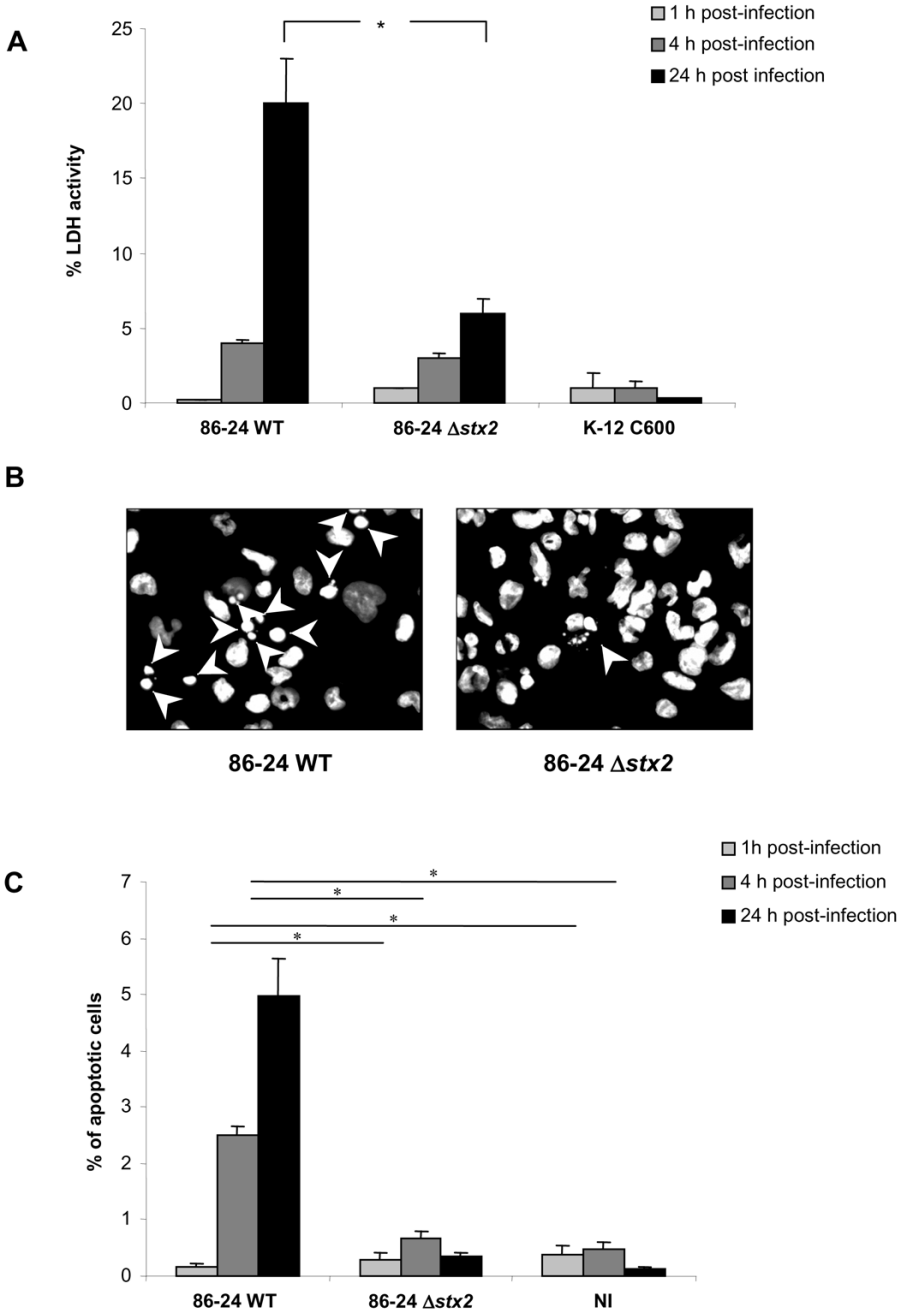
Induction of apoptosis in THP-1 macrophages infected by EHEC strains. Percentage of lactate dehydrogenase (LDH) activity at 1 h, 4 h, and 24 h post-infection (**A**). Fluorescence microscopy analysis of 86-24 WT and 86-24 Δ*stx*2 infected THP-1 macrophages stained with Hoechst at 24 h post-infection. Arrowheads indicate the fragmented nuclei of apoptotic cells (**B**). Quantification of apoptosis in infected THP-1 stained with Hoechst (**C**). Results are expressed as percentage of apoptotic cells numbered by microscopy observation. Results are mean ± SEM of four independent experiments. NI, non infected. Panel A) * 86-24 Δ*stx*2 significantly different from 86-24 WT at 24 h (*P*<0.05). Panel C) 86-24 Δ*stx2* and NI significantly different from 86-24 WT at 4 h and 24 h (*) (*P*<0.05).

Culture supernatants of infected macrophages were assayed for the presence of the cytoplasmic LDH to estimate the membrane integrity of the cells and the cytotoxic effect of EHEC strains. The amounts of LDH released were expressed as LDH activity recovered in the supernatant relative to LDH activity of total cell lysis defined as 100%. Infection of THP-1 cells with wild type 86-24 EHEC strain resulted in high release of LDH, which reached 20% at 24 h post-infection (Figure 5A). In contrast, the amounts of LDH released from Δ*stx*_2_-infected macrophages were significantly lower than those of cells infected with the 86-24 WT strain (*P*<0.05). Since Stx have been shown to induce apoptosis in epithelial, endothelial and monocytic cells [30], [31], we decided to perform fluorescence microscopy analysis of THP-1 nucleus stained with Hoechst. Unlike the 86-24 Δ*stx*_2_ mutant, macrophages infected with 86-24 WT for 24 h had fragmented nuclei, characteristic of apoptotic cells (Figure 5B). Quantifications of apoptotic cells are shown in Figure 5C. After a 4-h to 24-h infection, 86-24 WT increased the percentage of apoptotic cells, compared with cells infected with the Δ*stx*_2_ mutant or non infected (NI) cells (*P*<0.05). At 24 h, the percentage of apoptotic cells reached 5% of the remaining cells, compared to 0.3% and 0.1% with the Δ*stx*_2_ mutant and non infected cells, respectively (*P*<0.05).

**Figure 6:**
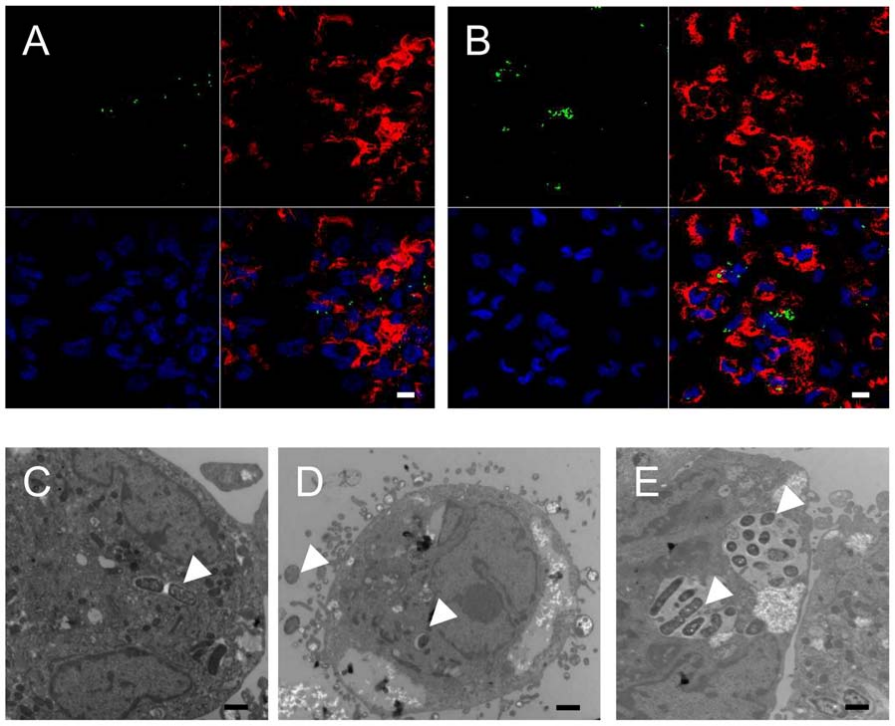
Survival and cytotoxic effects of EHEC strains within THP-1 macrophages. Confocal microscopy (**A** and **B**) and Transmission Electron Microscopy (TEM) (**C**, **D**, and **E**) analysis of THP-1 macrophages infected with EHEC strains. THP-1 macrophages were infected with the 86-24 WT strain (**A**, **C** and **D**) and with the 86-24 Δ*stx2* isogenic mutant (**B** and **E**) at 1 h post-infection (**A** and **C**) and at 24 h post-infection (**B**, **D** and **E**). For confocal micrographs, THP-1 macrophages were infected with GFP-positive 86-24 WT and 86-24 Δ*stx2* isogenic mutant (green color). The actin cytoskeleton of cells was stained with TRITC phalloidin (red), and DNA was stained with DAPI (blue). Arrowheads indicate bacteria within THP-1 macrophages. Scale bar = 10 µm (**A** and **B**). Scale bar = 1 µm (**C**, **D** and **E**).

Morphological data to confirm the cytotoxic effects and survival of EHEC wild type strain and the Δ*stx*_2_ mutant within THP-1 cells were obtained with confocal microscopy and TEM. One hour after infection, whatever the strain, many macrophages were infected with only a few bacteria present in small vacuoles (Figure 6A and C). At 24 h post-infection with 86-24 WT, cell layers were damaged and very few bacteria were found within macrophages (Figure 6D), suggesting that Stx production had driven the cells to apoptosis and release of intracellular bacteria into the antibiotic containing media. In contrast, THP-1 macrophages infected with the 86-24 Δ*stx_2_* mutant contained many bacterial clusters (Figure 6B and E), indicating that non-Stx-producing bacteria were able to replicate within macrophages; no morphological evidence of cytotoxicity was observed, confirming LDH release results.

## Discussion

EHEC O157:H7 are one of the most common pathogens involved in large outbreaks of severe gastrointestinal illnesses around the world [2], [6]. EHEC are implicated in a wide spectrum of clinical outcomes ranging from nonbloody diarrhea to HC and HUS. Stx, one of the major virulence factors produced by EHEC, plays an important role in the development of complications such as HUS [3]. To cause human illness, orally ingested EHEC must survive the acidic environment of the stomach [3]. However, recent studies evaluating the survival of an *E. coli* O157:H7 strain in a simulated *in vitro* model of human digestive tract, revealed a bacterial mortality in the stomach and duodenum. A bacterial growth was observed in the distal parts of the small intestine, suggesting that high levels of bacteria could be present in the ileum and in the colon [32]. EHEC are thought to colonize the colonic mucosa but do not invade deeper layers of the mucosa or spread systemically. Studies conducted using human intestinal IVOC have revealed a preferential tropism of EHEC for the FAE overlying ileal PPs, which is associated with a lack of colonic adhesion [12], [16], [17]. EHEC can induce A/E lesions on IVOC prepared from bovine ileum [16], but the principal site of colonization of EHEC O157:H7 is the lymphoid follicle-dense mucosa at the terminal rectum in the bovine host [24]. If EHEC target FAE, we hypothesized that the uptake of bacteria by M cells and underlying macrophages, which is observed for some pathogenic bacteria, may be the first step in EHEC translocation and subsequent toxin transport across the intestinal barrier.

It is now well documented that invasive members of the *Enterobacteriaceae* family, such as *Salmonella*, *Shigella* or AIEC, use M cells as the initial point of interaction with the host mucosa [18], [20], [22], [23], [33]. M cells transcytosis has been studied *in vitro* using a procedure to differentiate human intestinal epithelial cells (Caco-2) into M cells by co-culture with Raji B lymphocytes. This model exhibits morphological and functional characteristics of M cells, such as loss of microvilli, downregulation of brush-border enzymes, upregulation of particle transport, and enhanced translocation of pathogens [26], [27]. *Salmonella* translocation across this co-culture model was greater than in Caco-2 control monolayers, while translocation of EPEC, *Listeria monocytogenes* or *Clostridium difficile* was not enhanced in M cells compared to Caco-2 [18], [34]. We show here for the first time that EHEC strains, including O157:H7 and non-O157 serotypes, are able to significantly translocate through M cell monolayers compared to Caco-2 monolayers. A non pathogenic *E. coli* strain was not able to translocate at levels similar to that of EHEC strains. The high level of EHEC translocation through M cells is not a result of the loss of monolayer integrity, since the transepithelial electrical resistance of M cell monolayers was not modified after infection neither with EHEC strains 86-24 WT and EDL 933, nor with a non pathogenic *E. coli*. The Ussing chamber system is also a useful model of *ex vivo* intact organ culture to study mechanisms of bacterial translocation and the pathogenesis of enteric infections. Transmucosal uptake of EHEC strain 86-24 WT was confirmed and we observed high levels of bacterial translocation through murine ileal mucosa containing PP, whereas very few bacteria crossed the ileal mucosa in the absence of PP. *In vivo* ileal loop assays confirmed a specific targeting of EHEC to FAE overlying PP regions. Taken together, our *in vitro*, *ex vivo* and *in vivo* experiments demonstrate that EHEC strains target M cells on the surface of PPs in order to translocate across the epithelial barrier. Moreover, we describe for the first time that a two-hour contact with wild type EHEC induces hemorrhagic lesions in PPs in ileal loop assays, whereas such phenomenon is not found for AIEC strain LF82, an invasive *E. coli* pathovar known to target PPs [23]. A Stx-negative mutant is not able to induce bloodshot in ileal loop, which confirms the well known role of Stx in hemorrhagic lesions.

The molecular mechanisms that mediate the transport of bacteria across M cells remain unclear, and the bacterial effectors involved have not yet been totally identified. Intimin types might play a role in determining the pattern of colonisation and tissue tropism in the host, and intimin γ appears to restrict colonization of O157:H7 strains to human FAE [16]. Moreover, intimin γ binds β1-integrins, which are expressed on the apical surface of M cells [35]. Contrary to expectations, deletion of the *eae* gene in strain 86-24 did not alter translocation rates in our M cell model (Figure 2B) whereas the mutant was impaired in its capacity to adhere to epithelial intestinal cells (data not shown). Our results indicate that the LEE encoded genes may not promote translocation across M cells in EHEC. The long polar fimbriae (LPF), encoded by the *lpf* operon, play a key role in *Salmonella enterica* serovar Typhimurium [36] and in AIEC [23] to mediate bacterial interactions with M cells. The *lpf* operon was detected in an O91:H21 HUS associated strain, using a genomic subtractive hybridization procedure to identify virulence DNA sequences [37], and the complete genome sequence of O157:H7 strains revealed the presence of two *lpf* operons [38], [39]. Their expression is tightly regulated, and LPF were reported to be associated with adherence in EHEC O157:H7 to cultured epithelial cells [40]. However, deletion of one or both of the *lpf* operons in O157:H7 did not reduce FAE adhesion but enhanced colonization to small intestine, suggesting that LPF do not promote the targeting of PP in EHEC O157:H7 [41].

The enteric pathogens crossing the FAE are captured by resident macrophages and subepithelial dendritic cells in the dome of the lymphoid follicle [19]. In this study, we analyzed the entry, survival and proliferation of 17 EHEC strains and isogenic mutants in human THP-1 macrophages. We showed that EHEC were able to survive within macrophages for 24 h and to produce Stx, thereby inducing cell apoptosis of THP-1 macrophages and toxin release. Studies using human epithelial, endothelial and monocytic cell lines have shown that purified Stx induce apoptotic cell death *in vitro* [31]. The mechanism of Stx-induced apoptosis in the human myeloid leukaemia cell line THP-1 involves the increased expression of DR5 and TRAIL and activation of caspase-8 *via* a calpain-dependent mechanism through endoplasmic reticulum stress response [30], [31]. Furthermore, Stx encoding genes were seen to be up-regulated in intracellular bacteria [42].

Stx1 and Stx2 are able to cross polarized intestinal epithelial cells *via* a transcellular process and to remain biologically active after translocation, and it was shown that neutrophil transmigration enhanced the translocation of Stx, probably by opening a non-specific paracellular pathway across a polarized monolayer of T84 epithelial cells [43]. Here we propose an alternative pathway for Stx translocation *via* bacterial uptake by M cells and underlying macrophages. After bacterial uptake by M cells, Stx induce apoptosis in underlying infected macrophages, which leads to EHEC and toxin release in the *lamina propria*. In our experimental conditions, the average survival rate for the 86-24 wild type strain was only 51% because bacteria released into the antibiotic containing media were killed. In contrast *in vivo*, EHEC strains could be released in the *lamina propria*. We found that EHEC were able to replicate within THP-1 macrophages after 24 h but were killed after 48 h, unlike AIEC LF82 strain, which was still able to replicate within macrophages [28], [29]. More and more bacterial pathogens are observed to be able to translocate across the FAE *via* M cells, but their fate varies greatly depending on the pathogen. *Salmonella*, which are able to survive within macrophage, can spread throughout the organism, leading to bacteraemia and to systemic diseases. In contrast, infections due to *Shigella* or AIEC are restricted to the digestive tract. *In vivo*, EHEC could be eventually killed and therefore would not invade deeper layers of the mucosa, but the toxins released could gain access to the systemic circulation. The amount of toxin that translocates across intestinal epithelia is probably one of the most important factors in determining the development of systemic complications.

**Figure 7:**
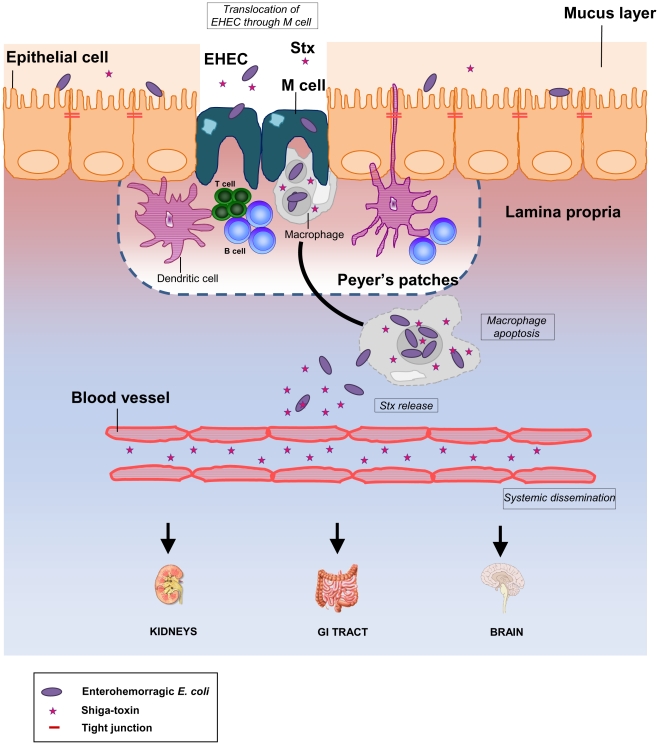
New working model for EHEC infection in humans. The diagram shows a monolayer of intestinal epithelial cells with EHEC infection in the lumen. Stx production occurs in the intestine. The bacteria cross the intestinal barrier through M cells. In the *lamina propria*, bacteria enter, survive, and produce Stx within resident macrophages. Following replication of bacteria in macrophages, extensive Stx production induces host cell death. Subsequently, released Stx could cross the downstream blood vessels to reach the kidneys, intestine, and brain. Damage to these organs results in serious life-threatening complications in humans.

As observed with *Shigella*, the infectious dose for *E. coli* O157:H7 estimated from outbreak data is considered to be as low as 1 to 100 organisms [3]. The targeting of bacteria to M cells and subsequent capture by macrophages could explain why only a small amount of bacteria would be sufficient to trigger disease, which determines, in part, the efficiency of the infection and the low infectious dose. We propose a new model for EHEC infection in humans (Figure 7) and suggest that bacteria cross the intestinal barrier through M cells overlying PPs. In the *lamina propria*, bacteria could enter, survive, and produce Stx within resident macrophages, inducing host cell apoptosis. In this case, however, bacteria would be eventually killed without causing bacteraemia. Subsequently, released Stx would cross the downstream blood vessels to reach the kidneys, intestine, and brain leading to severe disease in humans. The study of the relationships between interactions with M cells and the development of disease could help in designing novel therapeutic approaches to EHEC infection.

## Supporting Information

Figure S1 **Integrity of murine ileal mucosa determined by monitoring of FITC diffusion.** The mucosal integrity was monitored *ex vivo* in Ussing chambers by following FITC diffusion after infection with EHEC strain 86-24 WT and non pathogenic *E. coli* strain MG1655. Murine mucosa was isolated from the ileum, with Peyer's Patches (PP) (86-24 WT ▪ and MG1655 □ ) or without PP (86-24 WT • and MG1655 ○) placed in Ussing chambers during a 5-hour contact. One representative set of data is presented.
(TIF)

Figure S2 **Integrity of cell monolayers determined by trans-epithelial resistance (TEER) measurement.** TEER was monitored during a 5-hour infection in Caco-2 (A) and M cell monolayers (B), infected with EHEC strains 86-24 WT (Caco-2 ▴ and M cells ▵), and EDL 933 (Caco-2 • and M cells ○) or with a non pathogenic *E. coli* K-12 C600 (Caco-2 ▪ and M cells ◊).
(TIF)
