# Supplementary figures and images for: Interactions with M Cells and Macrophages as Key Steps in the Pathogenesis of Enterohemorragic Escherichia coli Infections
Source: PLoS One. 2011 Aug 17;6(8):e23594. doi: 10.1371/journal.pone.0023594 (PMC3157389; doi:10.1371/journal.pone.0023594)

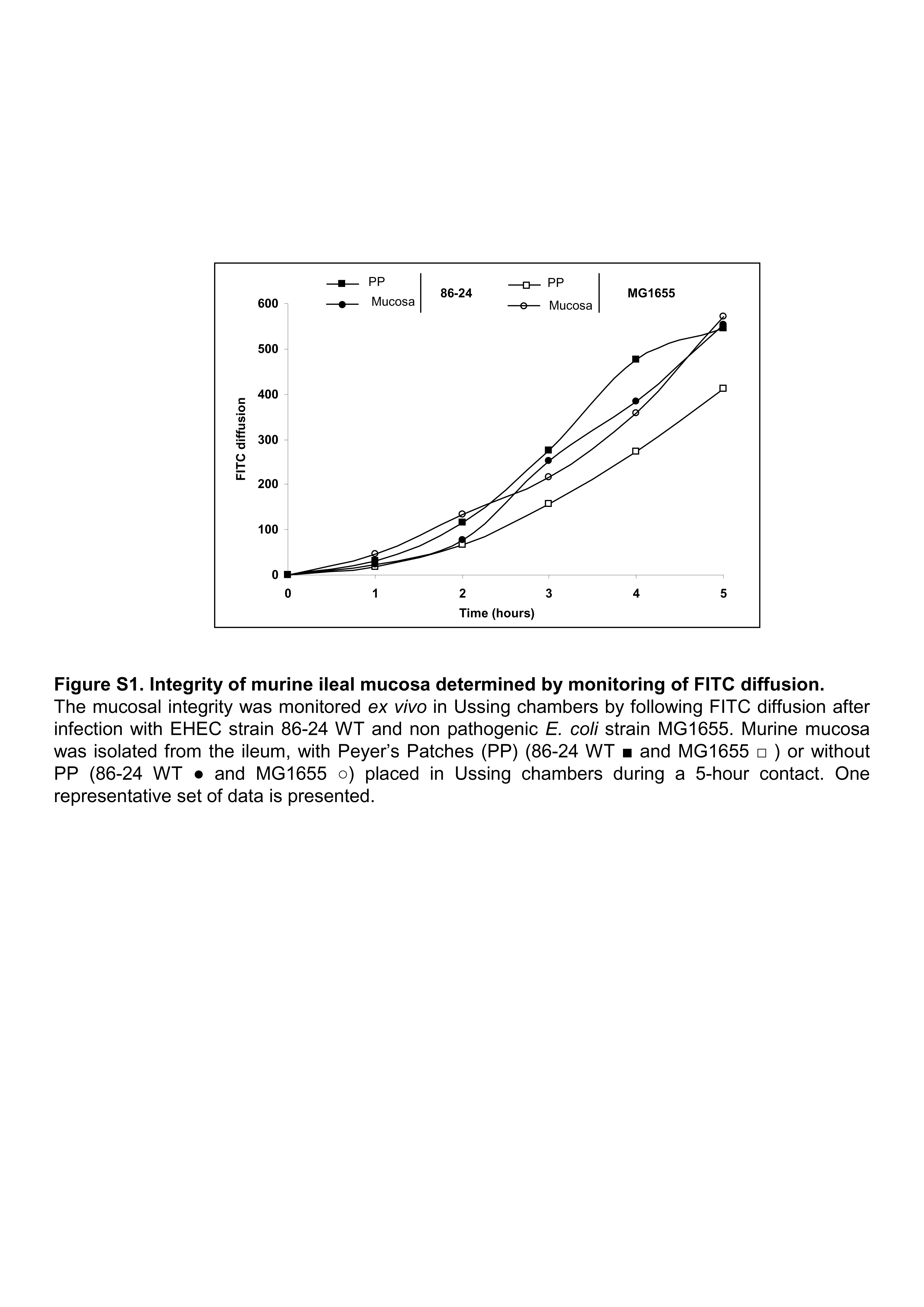

Supplement: Figure S1 — Integrity of murine ileal mucosa determined by monitoring of FITC diffusion. The mucosal integrity was monitored ex vivo in Ussing chambers by following FITC diffusion after infection with EHEC strain 86-24 WT and non pathogenic E. coli strain MG1655. Murine mucosa was isolated from the ileum, with Peyer's Patches (PP) (86-24 WT ▪ and MG1655 □ ) or without PP (86-24 WT • and MG1655 ○) placed in Ussing chambers during a 5-hour contact. One representative set of data is presented. (TIF) [file pone.0023594.s001.tif]

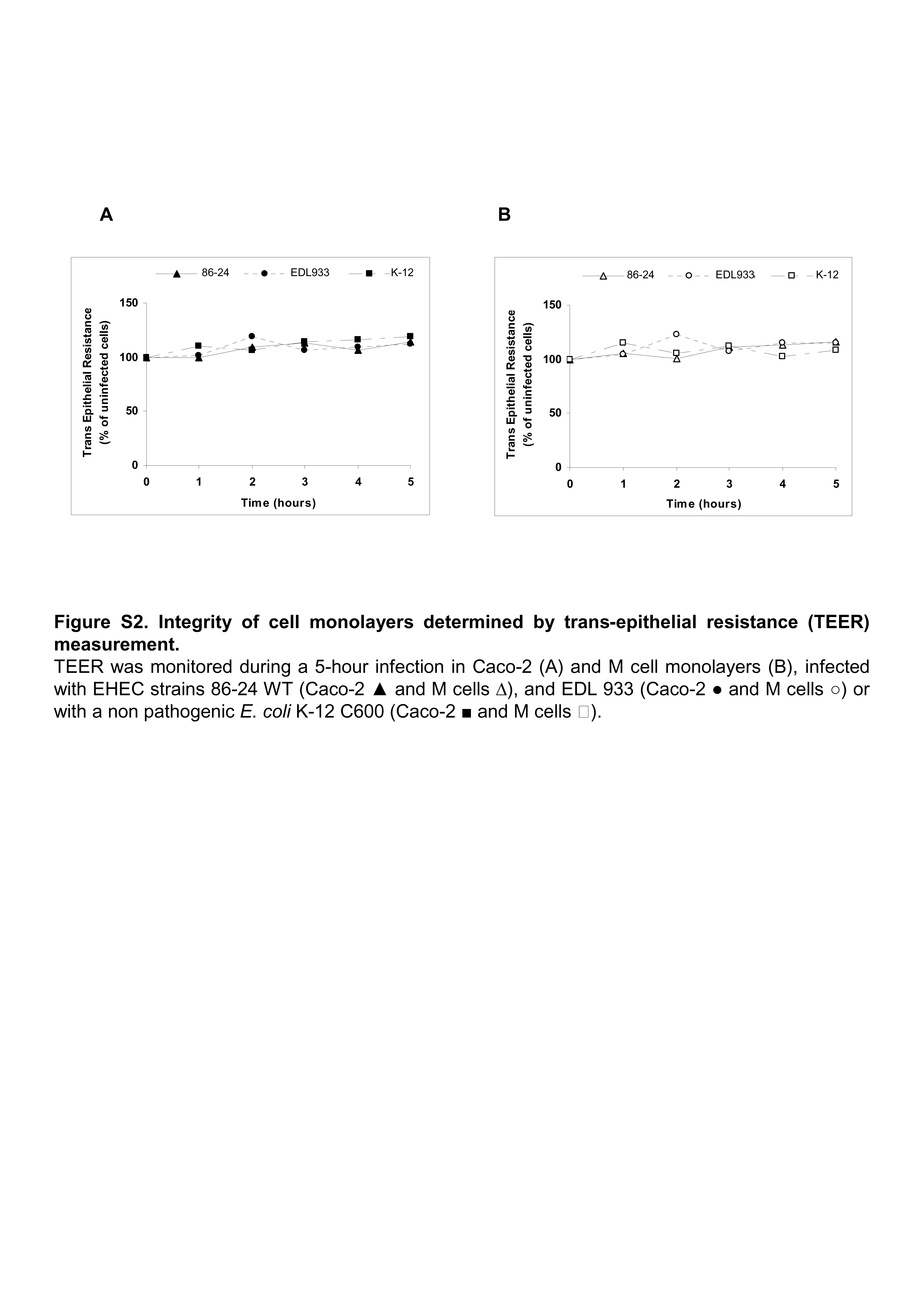

Supplement: Figure S2 — Integrity of cell monolayers determined by trans-epithelial resistance (TEER) measurement. TEER was monitored during a 5-hour infection in Caco-2 (A) and M cell monolayers (B), infected with EHEC strains 86-24 WT (Caco-2 ▴ and M cells ▵), and EDL 933 (Caco-2 • and M cells ○) or with a non pathogenic E. coli K-12 C600 (Caco-2 ▪ and M cells ◊). (TIF) [file pone.0023594.s002.tif]
